# Association between polymorphisms in genes encoding estrogen receptors (ESR1 and ESR2) and excreted bisphenol A levels after orthodontic bracket bonding: a preliminary study

**DOI:** 10.1186/s40510-018-0219-z

**Published:** 2018-07-02

**Authors:** Karla C. Horta, Guido A. Marañón-Vásquez, Mírian A. N. Matsumoto, Marília R. Moreira, Fábio L. Romano, Alberto Consolaro, Israel D. de Souza, Tamires A. V. Brigante, Maria E. C. Queiroz, Paulo Nelson-Filho, Erika C. Küchler

**Affiliations:** 1 Department of Pediatric Dentistry, Orthodontic Area, Ribeirão Preto Dental School, University of São Paulo, Ribeirão Preto, São Paulo Brazil; 2 Department of Pediatric Dentistry, Ribeirão Preto Dental School, University of São Paulo, Av. Do Café s/n, Monte Alegre, Ribeirão Preto, São Paulo 14040-904 Brazil; 3 Department of Stomatology, Bauru Dental School, University of São Paulo, Bauru, São Paulo Brazil; 4 Department of Chemistry, Ribeirão Preto School of Philosophy, Science and Literature, University of São Paulo, Ribeirão Preto, São Paulo Brazil

**Keywords:** Polymorphism, Bisphenol A, Orthodontics

## Abstract

**Background:**

Bisphenol A (BPA) is released from orthodontic composites used for bracket bonding. Genetic variations could modify the metabolism of this chemical within the organism. Considering that free BPA binds to estrogen receptors causing harmful effects to health, the present in vivo study aimed to evaluate the association between genetic polymorphisms in genes encoding estrogen receptors (*ESR1* and *ESR2*) and excreted BPA levels in orthodontic patients.

**Methods:**

Quantification of BPA levels in the urine of 16 patients was performed in a gas chromatograph mass spectrometer before (T0), at 24 h (T1), and 1 week (T2) after bracket bonding. DNA was extracted from saliva, and one genetic polymorphism in *ESR1* (rs2234693) and two in *ESR2* (rs4986938 and rs1256049) were analyzed by real-time PCR. Increases in BPA levels in the urine at T1 and T2 were grouped according to the genotype, and mean differences were compared by unpaired *T* test or Mann-Whitney test according to the normality of the data. The established alpha was 5%.

**Results:**

BPA levels increased significantly at T1 and T2. There were no statistically significant differences in the increases in BPA levels according to the genotype for any genetic polymorphism (*P* > 0.05), at neither 24 h nor 1 week after bracket bonding.

**Conclusions:**

The results suggested that there are no association between excreted BPA levels after bracket bonding and the evaluated genetic polymorphisms in *ESR1* and *ESR2*. Further research should be performed in order to confirm these results.

## Background

Bisphenol A (BPA; 2,2-bis [4-hydroxyphenyl] propane; CAS RN 80-05-7) is a synthetic industrial chemical employed to produce epoxy resins and polycarbonate plastics, which its production increases annually worldwide [1]. Exposure to BPA has been associated with some human diseases [2], even at lower doses [3, 4]. Human exposure occurs mainly orally by ingestion of food and/or beverages that were in contact with polycarbonate plastic [5, 6] or by contacting other synthetic products like thermal papers, medical devices, or dental materials [7]. It has been demonstrated that BPA can be released from bis-DMA or bis-GMA contained in resin-based materials used in dentistry [8, 9], including composites and adhesive systems used for bonding orthodontic appliances [10, 11].

Adverse effects of BPA are due to its xenoestrogenicity properties; the xenoestrogenicity aspects are the main reason of BPA adverse effects. Its chemical structure similar to natural estrogenic compounds (mainly 17-beta estradiol) confers to BPA the ability to bind to estrogen receptors (ERα and ERβ) [12, 13], expressing biologic effects similar to those induced by natural estrogens [14]. Therefore, BPA is considered an endocrine disruptor or a xenoestrogen substance due to the potential to disrupt estrogen-dependent normal physiology by deviating the hormonal homeostasis from the proper pathway [14, 15].

A previous investigation demonstrated that BPA is released from orthodontic composites used for bracket bonding [10]. In the study, the high variability among patients in the excreted BPA levels would show that there is an individual variability in the metabolism, absorption, and excretion of this chemical.

Differences in susceptibility to adverse effects of BPA may result from individual variability in the ability to effectively excrete this chemical [16]. Individual genetic background could be involved in this difference in susceptibility to BPA. Previous studies demonstrated that genetic variations modify the effect of BPA on tissues [17] and the metabolism of this chemical within the organism [18]. Remaining free BPA binds to estrogen receptors causing harmful effects to health. Currently, there are no studies available in the literature with the objective of exploring the influence of genetic variants in genes encoding estrogen receptors into the excreted levels of BPA.

For all mentioned above, we hypothesized that genetic polymorphisms in the genes encoding ERα (*ESR1*) and ERβ (*ESR2*) may alter (favor, hinder or modify) this binding, changing BPA kinetics and, consequently, the metabolism and the amount of excreted chemical. Therefore, the present study aimed to evaluate the association between polymorphisms in *ESR1* (rs2234693) and *ESR2* (rs4986938 and rs1256049), with BPA levels presented in the urine of orthodontic patients after bracket bonding.

## Methods

This study was approved by an ethics committee of the University of São Paulo, Ribeirão Preto, São Paulo, Brazil (protocol 34805914.9.0000.5419). Sixteen individuals (11 males and 5 females, aged from 10 to 19 years (mean age of 12.3 years)) were included in this research. Informed consent was obtained from patients and/or their guardians before clinical procedures.

Metallic brackets were bonding using the Transbond XT light-cure orthodontic adhesive system (3M Unitek, Monrovia, CA, USA). Data from BPA levels in the urine before bracket bonding (T0) and after 24 h (T1) and 1 week (T2) after this procedure were selected for the present study (patients’ data and methods were taken from a previously published research) [10]. The quantification of BPA in urine samples was performed by gas chromatograph coupled with mass spectrometer (GC-MS) liquid-liquid extraction [10].

Genomic DNA was obtained from saliva samples, as previously described [19]. The amount and purity of the DNA was determined by spectrophotometer (Nanodrop 1000; Thermo Scientific, Wilmington, DE, USA). The UCSC Genome Browser website was used to identify previously characterized genetic polymorphisms for each candidate gene (Table 1). Single-nucleotide polymorphisms (SNPs) in the ESR1 (rs2234693) and ESR2 genes (rs4986938 and rs1256049) were genotyped by real-time polymerase chain reactions (PCR) using the TaqMan assay (Step One Plus Real-Time PCR System, Applied Biosystems, Foster City, USA). Primers, probes, and the universal master mix were provided by Applied Biosystems (Foster City, CA, USA).

**Table 1:** Single-nucleotide polymorphisms studied

Gene	Locus	Reference sequence	Type of alteration	Base change (context sequence)	Global MAF
ESR1	6q25.1	rs2234693	Intron variant	AGC[**C**/T]GTT	0.4463/2235
ESR2	14q23.2	rs4986938	Intron variant, non-coding transcript variant, UTR variant 3 prime	AGC[C/**T**]TGT	0.2598/1301
ESR2	14q23.2	rs1256049	Intron variant, non-coding transcript variant, synonymous codon	CCG[C/**T**]ACT	0.1296/649

Source of information: dbSNP from: https://www.ncbi.nlh.nih.gov/snp/; http://genome.uscs.edu/; and, https://www.thermofisher.com

Bold indicates lower frequency allele

### Statistical analysis

Data from BPA levels (ng g^−1^) were examined for normal distribution (Kolmogorov-Smirnov test) and homogeneity of variance (Levene test). Mean differences between time points (baseline, 24 h, and 1 week) were verified by analysis of variance and Tukey post hoc tests. The increases in BPA levels were calculated for T1 (T1–T0) and T2 (T2–T0). These were grouped according to the genotype and compared by unpaired *T* test or Mann-Whitney test, according to the normality of the data. Similarly, mean differences for increases in BPA levels between male and female patients were analyzed. Statistical analysis was performed using GraphPad Prism 5.0a package (GraphPad, San Diego, CA, USA), and the established alpha was 5%.

## Results

Results of BPA levels in urine are presented in Table 2. In comparison with baseline (T0), BPA concentration in the urine increased significantly (*P* < 0.05) at T1 and T2 stages. The analysis of the increases in BPA levels by sex did not present statistically significant difference between male and female patients at 24 h (T1–T0) and 1 week (T2–T0) after bracket bonding (*P* > 0.05).

**Table 2:** BPA levels in urine

	Minimum	Maximum	Mean (SD)
BPA levels in the urine (ng g^−1^)			
Before bracket bonding (T0)	0.9	3.8	2.21 (0.99)^a^
24 h after bracket bonding (T1)	1.2	10.7	5.27 (2.62)^b^
1 week after bracket bonding (T2)	1.5	9.6	4.41 (2.14)^b^
Increases in BPA levels in the urine (ng g^−1^)			
Increases at 24 h (T1–T0)	0.3	8.1	3.06 (2.2)
Increases at 1 week (T2–T0)	0.3	6.8	2.21 (1.8)

Same letters indicate no statistically significant difference

**Fig. 1:**
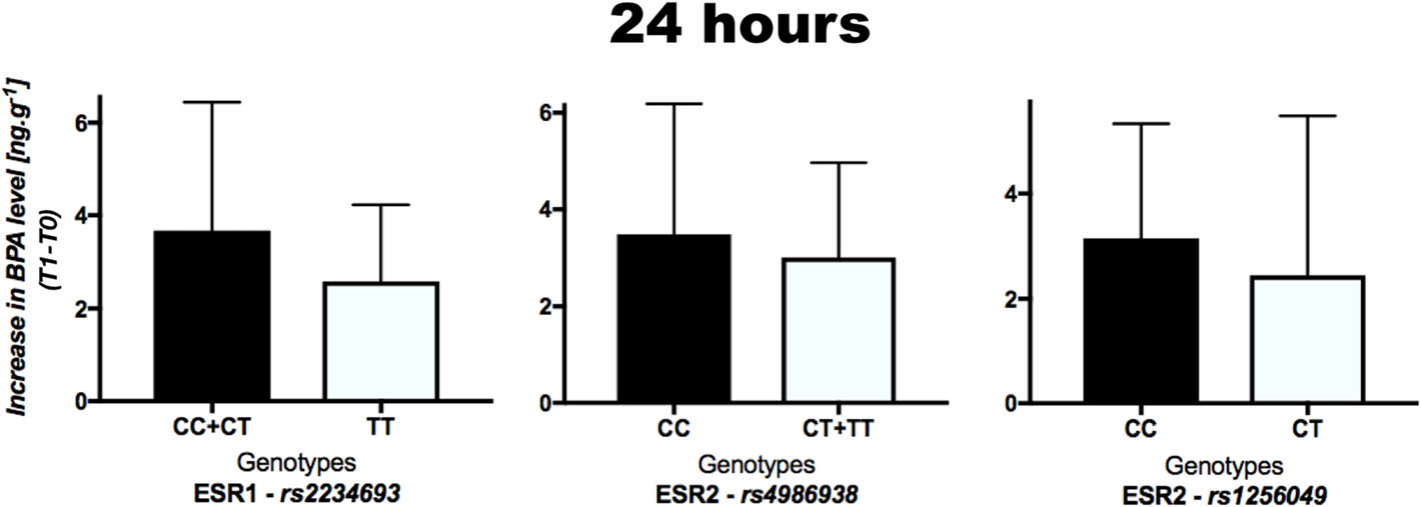
Means (SD) of the increase of BPA levels in urine 24 h after bracket bonding (T1–T0) according to genotype

Increases in BPA concentration in urine according to the genotype at 24 h after bracket bonding (T1–T0) are presented in Fig. 1. For the polymorphism rs2234693, the mean was 3.67 ng g^−1^ (SD 2.77) in CC+CT genotypes and 2.59 ng g^−1^ (SD 1.64) in TT genotype. For the rs4986938, the mean was 3.48 ng g^−1^ (SD 2.70) in CC and 3.02 ng g^−1^ (SD 1.95) in CT+TT genotypes. For the rs1256049, the mean was 3.15 ng g^−1^ (SD 2.19) in CC genotype and 2.45 ng g^−1^ (SD 3.04) in CT genotype. There were no statistically significant differences according to genotype in any polymorphism (*P* > 0.05).

**Fig. 2:**
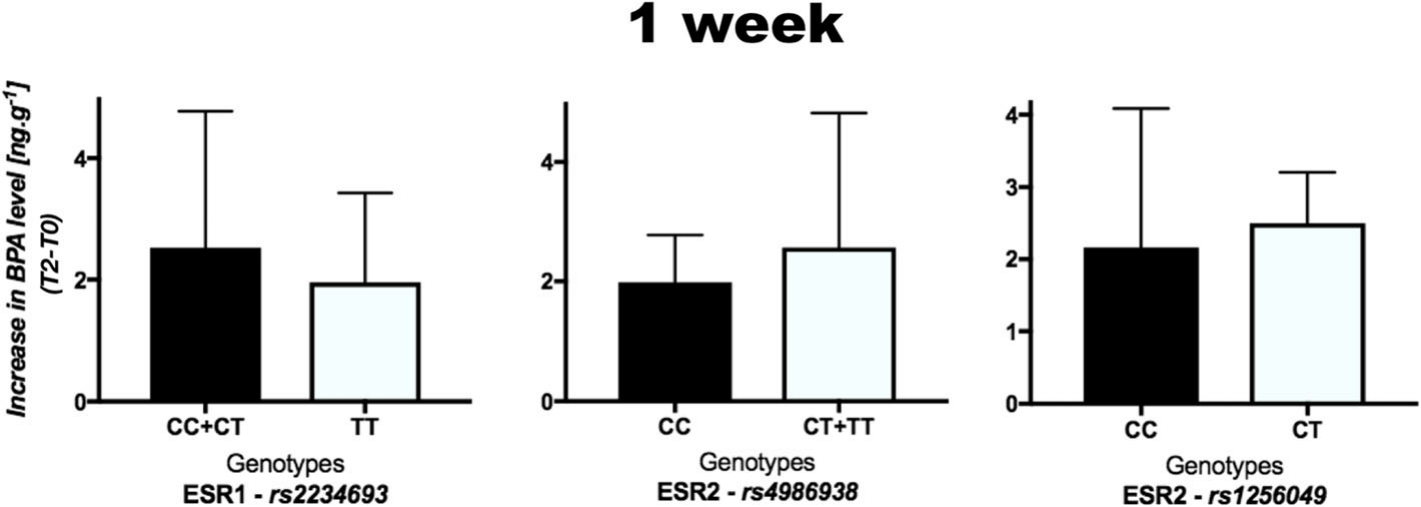
Means (SD) of the increase of BPA levels in urine 1 week after bracket bonding (T2–T0) according to genotype

Increases in BPA concentration in urine according to the genotype 1 week after bracket bonding (T2–T0) are presented in Fig. 2. For the polymorphism rs2234693, the mean was 2.53 ng g^−1^ (SD 2.24) in CC+CT genotypes and 1.96 ng g^−1^ (SD 1.47) in TT genotype. For the rs4986938, the mean was 1.98 ng g^−1^ (SD 0.79) in CC genotype and 2.57 ng g^−1^ (SD 2.25) in CT+TT genotypes. For the rs1256049, the mean was 2.16 ng g^−1^ (SD 1.92) in CC genotype and 2.5 ng g^−1^ (SD 0.71) in CT genotype. There were no statistically significant differences according to genotype in any type of polymorphism (*P* > 0.05).

## Discussion

The risks to public health related to the presence of BPA in dental materials have been studied by many research groups [8, 10, 20, 21]. However, to the best of our knowledge, this is the first study that aimed to explore the influence of genetic polymorphisms in genes encoding estrogen receptors, into the excreted BPA levels after dental treatment.

Data of the BPA levels in the urine used in the present study was previously published by Moreira et al. [10]. BPA levels in the urine indicate the amount of the chemical excreted [10]. Variability in this amount among patients could indirectly indicate also variability in the amount of free BPA in the body.

Alterations in metabolism and routes that this chemical follows within the body may cause variations in the amount absorbed and/or excreted of BPA. After an almost immediate absorption from the gastrointestinal tract [22, 23], BPA is metabolized mainly by glucuronidation in the liver [24, 25], a process that facilitate its inactivation and elimination. So, most of the BPA is conjugated from a bioactive estrogenic form to a non-estrogenic form and later eliminated; however, some unconjugated BPA remains in the circulation [26]. BPA-derived compounds, upon undergoing the conjugation process, do not bind to the estrogens receptors and cannot be accumulated [27]. Alterations in the conjugation processes or an excess of action of the beta-glucoronidase leads to a deficient detoxification of the BPA, reintroducing itself in the circulation and causing adverse effects to the health [28, 29].

Resin-based dental materials contain BPA derivatives that can release this chemical in its raw form due to the incomplete monomer polymerization or as a result of material degradation [30, 31]. Previous studies [21, 32, 33] have reported that the levels of BPA released by this type of dental material are below the recommended limit, but these low doses are considered as an additional source of exposure and could cause adverse effects [3]. In the present study, BPA levels in the urine of the patients were relatively low and far below established limits, similar to other studies conducted with orthodontic materials [30, 34–37].

Data from a previously published research showed an increase in BPA levels in patients’ urine [10]; however, although all patient received the same amount of resin, the authors were able to note a high variability in these levels between patients. This fact is probably related to the individual ability to metabolize BPA. Differences in the susceptibility of people to adverse effects of BPA may have an environmental (exposure-related cause) and/or genetic explanation [16].

Genetic variability could be the cause of this difference in susceptibility to BPA. It has been reported that genetic polymorphisms in UGT2B15, UGT1A9, and UGT1A1, genes related to BPA metabolism contribute to the glucuronidation variability of this chemical on the breast and liver [18] and that the influence of functionally relevant polymorphic UGT2B15 alters the BPA concentration in blood [38].

We hypothesized that genetic variations in genes encoding ERα (*ESR1*) and ERβ (*ESR2*) may alter (favor, hinder, or modify) this binding, changing BPA kinetics and consequently the metabolism and amount of excreted chemical. Thus, we chose *ESR1* and *ESR2* as candidate genes to perform a preliminary investigation to evaluate if there was an association between polymorphisms and the BPA levels presented in the urine of orthodontically treated patients after bracket bonding. The null hypothesis was accepted; our results did not present any statistical significant difference between the increases in BPA levels according to genotype for any polymorphism.

It is important to mention that our small sample size could be responsible for a type II error. Further researches with higher sample sizes and with more periods evaluated should be carried out to confirm this hypothesis. In the present study, the periods of 24 h and 1 week after bonding were chosen, because the quantity of BPA levels were statistically different from baseline levels, different from the 1-month period that did not show this difference as previously published [10]. Perhaps, the periods with 2 or 3 weeks should be assessed due to the broad variance in results previously published [34–36].

Likewise, it is suggested to replicate this study in a sample stratified by sex and age. Despite the fact that estrogen receptors have a wide distribution in female tissues and organs, this could not have influenced our results. The analysis of the increases in BPA levels by sex did not present statistically significant difference between male and female patients.

Young patients represent the majority of patients in orthodontic clinic. Therefore in this preliminary study, we focused on young patients due the fact that teenagers exposed to high BPA levels are related to some diseases and conditions such as obesity [39], increased risk of low-grade albuminuria [40], carotid intima-media thickness [41], polycystic ovary syndrome [42], and insulin resistance [43].

Although the functional implications of the SNPs rs2234693, rs4986938, and rs1256049 have not been fully determined, we hypothesized that these intronic variants could modify the binding of BPA to ERs altering its metabolism and subsequent excretion, since some previous studies reported an association with different conditions [44–46] suggesting that these SNPs have an important biological role. Studies of genetic differences in substances metabolic pathways that can affect individual responses are in early stages and should be performed in many areas of dental research in order to establish a future field of personalized dentistry.

## Conclusions

No association between polymorphisms in genes encoding estrogen receptors (*ESR1* and *ESR2*) and excreted BPA levels was found in orthodontic patients after bracket bonding.

## Abbreviations

bis-DMA: Bisphenol A, dimethacrylate
bis-GMA: Bisphenol A, glycidyl dimethacrylate
BPA: Bisphenol A
ERα: Estrogen receptor Alfa
ERβ: Estrogen receptor Beta
ESR1: Estrogen receptor Alfa gen
ESR2: Estrogen receptor Beta gen
GC-MS: Chromatograph coupled with mass spectrometer
SNP: Single-nucleotide polymorphism
